# Therapeutic drug monitoring of lithium therapy at a psychiatric hospital in Namibia

**DOI:** 10.11604/pamj.2023.45.78.39862

**Published:** 2023-06-07

**Authors:** Bonifasius Siyuka Singu, Jessie Horases, Roger Karel Verbeeck

**Affiliations:** 1 School of Pharmacy, Faculty of Health Sciences and Veterinary Medicine, University of Namibia, Windhoek, Namibia

**Keywords:** Lithium, therapeutic drug monitoring, psychiatry

## To the editors of the Pan African Medical Journal

Psychiatric disorders in Africa are generally under-studied and under-reported in comparison to other parts of the world [1]. A scoping review of 36 studies across 12 African countries reported a lifetime prevalence of various psychiatric disorders such as mood disorders (3.3-9.8%), anxiety disorders (5.7-15.8%), substance use disorders (3.7-13.3%), and psychotic disorders (1.0-4.4%) [2]. Treatment guidelines consider lithium therapy as first-line maintenance therapy against bipolar disorder, mania, and acute bipolar depression [3]. Although lithium is effective against psychiatric conditions, its toxicity profile and narrow therapeutic range make therapeutic drug monitoring (TDM) necessary [4]. Since lithium is eliminated via the kidneys, dose adjustment is recommended in patients over 40 years old [5]. The therapeutic range for lithium as maintenance therapy is considered by some to be 0.6-0.8 mmol/L and 0.6-1.2 mmol/L by others, and the maintenance therapy concentrations are between 0.8-1.2 mmol/L [3]. Although lithium TDM guidelines are well documented, several reports indicate poor adherence [6]. The aim of this study was to report on the TDM of lithium at the Psychiatry Department of Windhoek Central Hospital (PD-WCH), Namibia. The PD-WCH is the national referral psychiatry facility and serves both out and in-patients (with a bed capacity of 140).

This study was a retrospective review of clinical records of patients who received lithium therapy over a five-year period (2016-2021) at the PD-WCH. Data extracted were: duration of therapy, age, sex, dose, number of admissions, plasma concentrations of lithium, creatinine, thyroxine (T3 and T4), and sodium. Approval for this study was obtained from the Namibian Ministry of Health and Social Services (MoHSS) Research Ethics Committee; reference number JLQH2021.

**Figure 1:**
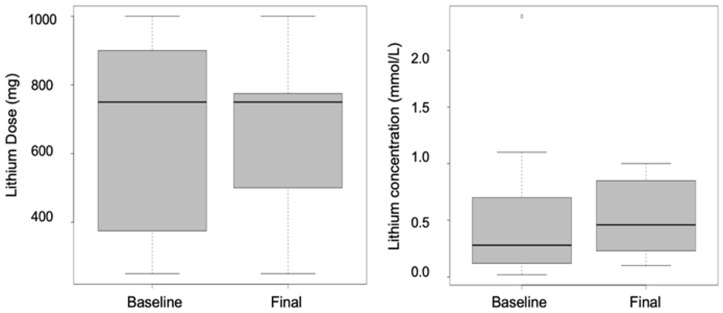
median lithium dose and lithium plasma concentrations at baseline and after adjustments (n=11)

A total of 12 records were retrieved, but one was missing relevant treatment information. Five (5) of the 11 patients were female; the age range of the 11 was 20-59 years (Table 1). Only one patient had body weight recorded in their records. Records show that patients were on lithium therapy for a period of 2 months to 6 years, during which they were admitted 0-6 times as a result of relapse in psychiatric episodes. Of the 11 patients included in this study, 5 received lithium for bipolar mood disorder, 4 had bipolar schizoaffective disorder and 2 for the bipolar schizophrenic disorder. The 11 patients had 1-4 plasma lithium concentrations collected 12 hours post-dose for TDM, with 0-3 dose adjustments reported during their recorded periods of treatment. Although baseline and final (first and last recorded doses received by the patient, respectively) median dose and range were similar (median: 750 mg; range: 250-1000 mg), the final median plasma lithium concentration (0.5 mmol/L) was higher than the baseline (0.25 mmol/L) with range values of 0.03-1.37 and 0.18-1.00 mmol/L, respectively (Figure 1) - the boxes represent the 25^th^, 50^th^ and 75^th^ percentiles and the whiskers represent the range (minimum and maximum) for doses and concentrations (n=11). Baseline concentration is the first lithium plasma concentration that was recorded while the final concentration is the last recorded lithium plasma concentration for the individual patient. The baseline dose is the first lithium dose that was recorded while the final dose is the last recorded lithium dose for the individual patient. T3 levels ranged from 1.54- 4.37 pmol/L (reference: 3.1-6.8 pmol/L) while T4 plasma concentrations ranged from 7.6-15.04 pmol/L (reference: 12-22 pmol/L). Creatinine plasma concentrations ranged from 63-113 µmol/L (reference: 60-110 µmol/L), and sodium plasma concentrations were 136-143 mmol/L (normal: 135-145mmol/L).

**Table 1:** demographic characteristics of psychiatric patients who received lithium therapy at Windhoek Central Hospital for the years 2016-2021 (n=11)

Patient	Sex	Age (years)	Diagnosis	Length of treatment (years)	Number of admissions	Number of dose adjustments	Number of lithium readings
A	M	59	Bipolar mood disorder Type I	2	0	1	3
B	M	20	Bipolar schizoaffective disorder	> 2	1	1	4
C	F	40	Bipolar mood disorder	5	4	2	3
D	F	38	Bipolar mood disorder Type I	3	2	0	3
E	M	27	Bipolar schizophrenic disorder	> 0.3	2	3	3
F	F	46	Bipolar schizoaffective disorder	> 5.6	6	1	2
G	F	43	Bipolar schizophrenic disorder	1.25	1	2	2
H	F	30	Bipolar schizoaffective disorder	> 3	3	3	4
I	M	56	Bipolar mood disorder Type I	0.2	3	0	2
J	M	40	Bipolar mood disorder	> 4	2	0	2
K	M	58	Bipolar schizoaffective disorder	1	2	2	1

> means patient still on lithium therapy by the time data was collected

This review found that there was no difference in median baseline and final lithium doses. In addition, although the final plasma lithium concentrations were higher than the baseline, they were in the subtherapeutic range. Creatinine and sodium concentrations were normal in all patients, while T3 and T4 levels were low in some cases. Although samples were collected 12 hours post-dose as stated by guidelines, it was not carried out weekly after initiation, after each dose change, and then every 3 months after concentrations are stable as recommended in some guidelines [7]. The low-frequency sample collection resulted in the dose during treatment remaining unchanged, subtherapeutic plasma lithium concentrations, and a frequent hospitalization rate averaging once per year. To avoid toxicity due to overdosing, clinicians take caution by selecting the lowest recommended dose, but such dosing is likely to be subtherapeutic if there is no subsequent dose adjustment in accordance with reported lithium concentrations. Similar TDM studies of other narrow therapeutic indexes drugs such as phenytoin, valproic acid, and gentamicin at this same hospital have also reported poor achievement of target plasma drug concentrations [8,9]. Laboratory costs, shortage of equipment, and lack of expertise in pharmacokinetics required for correct planning, interpretation of levels, and dose adjustment are some of the reasons why TDM may not be routinely and correctly practiced, especially in a developing country such as Namibia [10]. The main limitation of this retrospective study was that patient records did not include patient body weight readings which made it impossible for the assessment of creatinine clearance as an estimate of kidney function. Another limitation was that the small sample size could not allow for population statistical inference.

## Conclusion

Results from this study show that TDM of lithium therapy is rarely practiced at this facility. Patients received subtherapeutic lithium dose as evidenced by subtherapeutic plasma lithium concentrations and no apparent biochemistry signs of adverse drug reactions (as the dose was too low).
